# Permanent cardiac pacing: first Congolese experiment

**DOI:** 10.11604/pamj.2015.20.381.5803

**Published:** 2015-04-16

**Authors:** Stéphane Méo Ikama, Jospin Makani, Xavier Jouven, Gisèle Kimbally-Kaky

**Affiliations:** 1 Service de Cardiologie et Médecine Interne, CHU de Brazzaville, B.P. 2234 Brazzaville, Democratic Republic of Congo; 2 Service de Cardiologie, Hôpital Européen Georges Pompidou, Paris, France

**Keywords:** Atrioventricular block, pacemaker, quality of life, Congo

## To the editors of the Pan African Medical Journal

In Africa, except the Maghreb countries and South Africa [1, 2], the cardiac pacing activity is poorly developed for economic reasons and due to shortage of qualified personnel. Also, most establishments are done by means as of medical evacuations, or via the missions of cardiac pacing to humane aiming and formation [3]. This is the case of Congo, which benefitted from a French cardiac pacing mission in January 2012. In this preliminary study, we report about the Congolese experiment by presenting the profile of the first patients who underwent pacemaker implantation in Congo. The study was a longitudinal and descriptive one carried out in the service of cardiology and the surgical unit the University Hopsital of Brazzaville from January to September 2012. On an initial waiting list of 20 patients, eight died before the mission took place, and twelve answered the call favorably, but four did not come. The study related to eight patients who underwent pacemaker implantation during a French cardiac pacing mission. The effect of the pacemaker on the symptoms and the quality of life of the patients were evaluated according to the CDC-HRQOL4. Mean age of patients was 70.4 ± 6 years. There were four men and four women. Five patients were living in Brazzaville and three came from others departments. Functional signs consisted of: dyspnea (7 cases), syncope (3 cases), syncopal equivalents (2 cases). One patient was asymptomatic. The average heart rate was 37.4 beats per minute (range: 27 and 49 beats). ECG showed: a complete atrioventricular block (6 cases), an atrioventricular block of second degree (2 cases). Cephalic venous access was used in six patients and subclavian in two others. A cardiac arrest occurred in three patients, cardiac massage was performed, in the absence of temporary stimulation and isoprenaline. All patients notified improvement of symptoms and quality of life during the first month of implantation. No complication was observed during the nine months of follow-up. The management of cardiac rhythm and conduction disorders remains difficult in Sub-Saharan Africa. Indeed, if the fundamental problems are that of the insufficiency of the technical support center, the often modest social conditions of the populations constitute also a constraint to accessibility to care. As regards cardiac stimulation, the complete atrioventricular block or of high degree constitutes of it the principal indication [4, 5]. The pacemaker implantation, in rather simple rule, can sometimes be peppered with incidents during intervention, in particular in our context. A cardiac arrest can occur, favoured at the same time by the importance of the bradycardia, the absence of temporary stimulation and positive chronotropic drugs (Isoprenaline). It is in addition necessary to underline the long period of waiting between the moment when the indication of installation of pacemaker is retained and its effective realization. In Africa, much longer evolutions are often observed among patients carrying complete atrioventricular block. The risk of sudden death being always possible, this one could enamel the spontaneous evolution of some of our defaulted patients. The beneficial effect of the pacemaker on the improvement of the symptoms and the quality of life of the patients, raised in our study, was reported by other authors [6].

## Conclusion

The pacemaker implantation is now a reality in Congo. This preliminary study showed the benefit of the pacemaker implantation on the symptoms and the quality of life of the patients. Also, this activity should be continued in order to decrease the health expenditure subsequent to medical evacuations costs.
